# Ranking Single Fluorescent Protein-Based Calcium Biosensor Performance by Molecular Dynamics Simulations

**DOI:** 10.1021/acs.jcim.4c01478

**Published:** 2024-12-27

**Authors:** Melike Berksoz, Canan Atilgan

**Affiliations:** Faculty of Engineering and Natural Sciences, Sabanci University, Istanbul 34956, Turkey

## Abstract

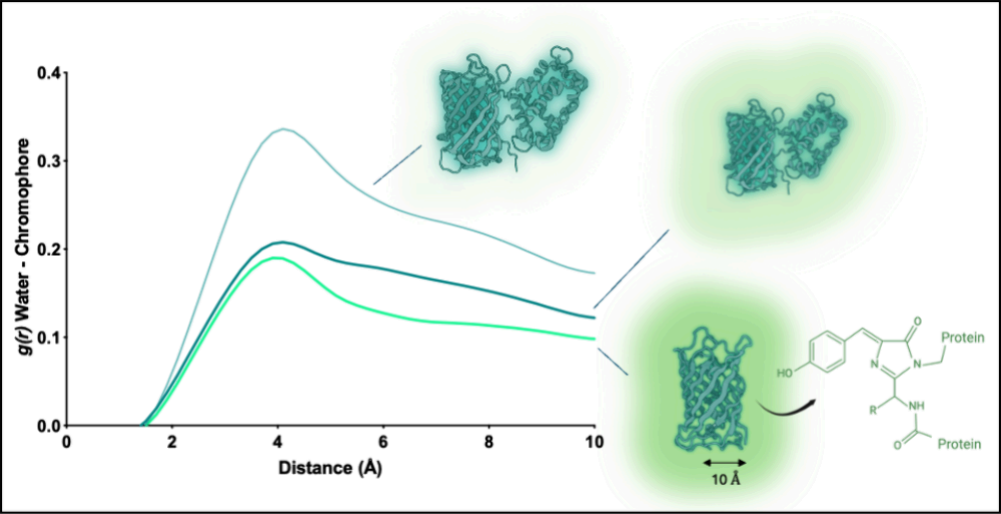

Genetically encoded fluorescent biosensors (GEFBs) have become indispensable tools for visualizing biological processes *in vivo.* A typical GEFB is composed of a sensory domain (SD) that undergoes a conformational change upon ligand binding or enzymatic reaction; the SD is genetically fused with a fluorescent protein (FP). The changes in the SD allosterically modulate the chromophore environment whose spectral properties are changed. Single fluorescent (FP)-based biosensors, a subclass of GEFBs, offer a simple experimental setup; they are easy to produce in living cells, structurally stable, and simple to use due to their single-wavelength operation. However, they pose a significant challenge for structure optimization, especially concerning the length and residue content of linkers between the FP and SD, which affect how well the chromophore responds to conformational change in the SD. In this work, we use all-atom molecular dynamics simulations to analyze the dynamic properties of a series of calmodulin-based calcium biosensors, all with different FP–SD interaction interfaces and varying degrees of calcium binding-dependent fluorescence change. Our results indicate that biosensor performance can be predicted based on distribution of water molecules around the chromophore and shifts in hydrogen bond occupancies between the ligand-bound and ligand-free sensor structures.

## 1 Introduction

Genetically encoded fluorescent biosensors (GEFBs) are widely used as molecular imaging tools. Virtually any analyte can be traced in the transgenic cells expressing the fluorescent sensor, which binds selectively to the analyte of interest. Two main classes of GEFBs are Förster Resonance Energy Transfer (FRET)-based sensors and single fluorescent protein (FP)-based sensors. FRET sensors are composed of two FPs and a sensing protein, which undergoes a large enough conformational change upon ligand binding that effectively alters the distance between the two FPs, allowing for energy transfer between their fluorophores, detectable as a change in fluorescence intensity.^1^ Single FP biosensors are constructed by the fusion of a single FP and a sensory domain. Circular permutation of the FP allows for the insertion of a sensing protein near the chromophore.^2^ Fluorescence is modulated by ligand binding/unbinding and the subsequent changes in the hydrogen bonding pattern around the chromophore.^3,4^ Excitation with light of suitable wavelength leads to acidification of phenolic hydrogen of the chromophore, which is transferred via a network of hydrogen bonds to a nearby glutamate residue. The remaining anionic phenolate moiety displays higher fluorescence than the neutral form.^5,6^ Therefore, a shift in the hydrogen bond pattern around the chromophore due to a conformational change directly affects the fluorescence output of the sensor.

**Figure 1:**
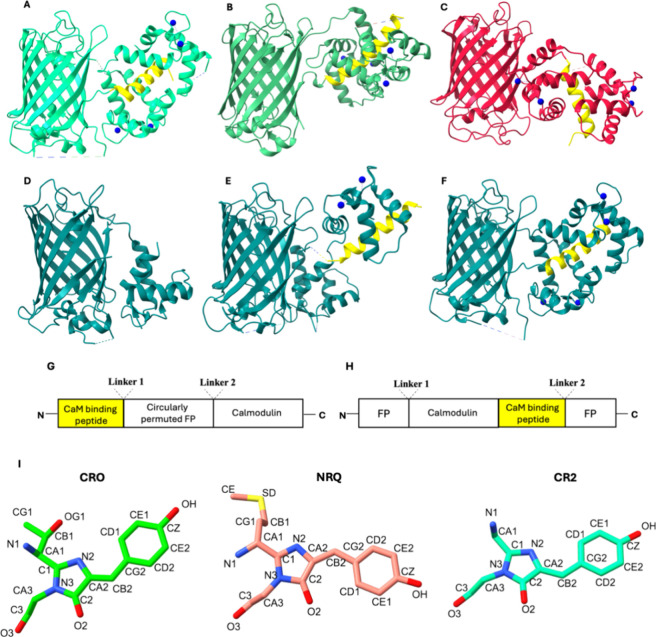
(A) Four Ca^2+^-bound jGCaMP8 (PDB: 7ST4). (B) Four Ca^2+^-bound NCaMP7 (PDB: 6XW2). (C) Four Ca^2+^-bound RCaMP1a (PDB: 3U0K). (D) Calcium-free GCaMP2 with a partially resolved CaM domain (PDB: 3EKJ). (E) Two Ca^2+^-bound GCaMP2 (PDB: 3O77). (F) Four Ca^2+^-bound GCaMP2 (PDB: 3EVR). α-Helical peptide associated with the CaM domain is shown in yellow in all cases. (G) Schematic sequence of jGCaMP8, RCaMP1a, and GCaMP2. (H) Schematic sequence of NCaMP7. (I) Chromophore types. CRO, NRQ, and CR2. Only the heavy atoms provided in the PDB structures are shown; carbons are colored according to the fluorescence color of the respective chromophore. Atom types are taken from CHARMM36 force field topologies.

Genetically encoded calcium indicators (GECIs) are the earliest and most widely studied single FP biosensors, given the important role of calcium in neural activity and as a secondary messenger in intracellular signaling.^7,8^ As of 2024, the largest group of fluorescent biosensors is by far the calcium sensors^9^ (Figure 1). Two main strategies are used in the design of GECIs: (i) a circularly permuted FP is fused to calmodulin (CaM) at its C terminus and to a CaM-binding peptide at its N terminus (Figure 1G) or (ii) an intact CaM-peptide moiety is inserted into the FP sequence (Figure 1H). The α helical peptide hydrophobically interacts with CaM and “holds” the two domains together.^10^ Inside the FP’s β barrel, the chromophore, which is responsible for the spectral properties, exists in an equilibrium of neutral and anionic forms.^11^ Ca^2+^ binding triggers a conformational change in CaM, which translates to a shift in the p*K*_a_ of the chromophore and moves the equilibrium toward the anionic form.^4^ In some cases, fluorescence lifetime and quantum yield may also be altered as a result.^12^ For most GECIs, the anionic chromophore is the brighter form; therefore, Ca^2+^ binding causes a detectable increase in fluorescence intensity. Structural studies of GCaMP2 showed that in the calcium-saturated form, the two domains of CaM wrap around the M13 peptide, effectively blocking the solvent access to the circular permutation (cp) site, which helps keep the chromophore in its anionic-fluorescent state.^13^ In fact, reduced solvent access to the chromophore may be a common feature of biosensors in the high fluorescent state.^14^ The two-calcium-bound GCaMP2 has an extended structure where the N and C terminal domains of CaM are distal to each other, and M13 interacts only with the N terminal domain carrying the two calcium ions (Figure 1E). To date, there is only one published calcium-free structure of GCaMP2 where only the FP and the N terminus domain of CaM are resolved, while the M13 peptide and the C terminal domain are missing (Figure 1D). The *Holo* structure of other GCaMP-like GECIs reveals a similar organization, although the positioning of the CaM-peptide moiety relative to the FP domain differs depending on the circular permutation strategy and linker length (Figure 1A–C).

Biosensor performance is generally ranked based on a few biophysical features; ligand binding affinity *K*_d_, response rate, extinction coefficient, quantum yield, and ligand binding-dependent change in fluorescence signal amplitude Δ*F*/*F.*^15−17^ While sensor performance is inherently tied to quantum mechanical factors stemming from the changes in the electronic environment of the chromophore, treatment of the system at that level of theory might allow for focusing on a single system with the currently available computers. Such an approach would already be an endeavor since it is no trivial task to optimize for the chromophore environment with the right number of atoms in a truncated system. However, this is at odds with our aim to lay out a computationally tractable pipeline that allows for comparing several systems and provide a prediction of their relative fluorescence, which would require a classical treatment that takes the whole system into account. In fact, Δ*F*/*F* may be a suitable property to deduce from molecular dynamics (MD) simulations where the Ca^2+^ binding-induced conformational changes can be sampled within reachable time scales.^18−21^ High Δ*F*/*F* might imply a large conformational change upon ligand binding in the sensory domain (SD) that greatly changes the local environment and the p*K*_a_ of the chromophore; moreover, a few key residues at the interface between the SD and FP may be especially effective in communicating the conformational change to the chromophore. To correlate the protein dynamics obtained from MD simulations with fluorescence, structural requirements for the chromophore should be defined. Crystal structures of various biosensors revealed a few key properties of the ON state sensors: (i) the anionic chromophore is stabilized by a direct or a water-mediated hydrogen bond with a nearby donor residue located on the FP, linker or SD, (ii) the two rings of the chromophore are coplanar as in parental FPs, and (iii) the opening at the cp site where the chromophore protrudes is partially occluded by the SD, which reduces the solvent exposure and helps preserve the anionic high fluorescent state.^22−26^

In this work, we analyze a series of GECIs with varying ligand binding-dependent Δ*F*/*F* values with the motivation to reveal the allosteric mechanism of conformational change and its effect on the chromophore environment using classical all-atom MD simulations. We have chosen green GECIs, GCaMP2,^13,27^ NCaMP7,^28^ and jGCaMP8^29^ and red GECI RCaMP1a.^30^ The differences in their parental FPs, chromophore types, calcium binding-dependent Δ*F*/*F* values, CaM-binding peptides, and circular permutation strategies have motivated us to choose these sensors for a detailed investigation to propose a unified working mechanism that may also be predictive for new designs. Calcium-saturated crystal structures of all these sensors are available in the PDB (Figure 1A,B,C,F). GCaMP2 is the earliest designed sensor in our set. It is an improved version of the original GCaMP, the first single FP-based Ca^2+^ indicator, and is derived from circularly permuted EGFP (Figure 1D–F). RCaMP1a is derived from the fusion of circularly permuted mRuby and CaM-M13 (Figure 1C). In NCaMP7, the CaM-M13 moiety is inserted into mNeonGreen (Figure 1B). This type of design was demonstrated to be advantageous in terms of calcium sensitivity and dynamic range.^28,31^ The most recent and superior one among the four sensors is jGCaMP8. Initial variant “JGCaMP8.410.80”, whose crystal structure was resolved, has a Δ*F*/*F* of 75.^29^ It is composed of cpGFP, CaM, and the CaM-binding peptide from endothelial nitric oxide synthase (ENOSP), which has a considerably different sequence than the M13 peptide (Figure S1).

As part of the design of single FP-based sensors, the opening created near the cp site on FPs exposes the chromophore to nearby ionizable residues as well as solvent molecules, which may protonate the anionic phenyl group and quench the fluorescence. Furthermore, water access into the β barrel may disrupt the hydrogen bonding network, which is crucial for the FP to function. We test the degree of exposure to solvent molecules by measuring the solvent-accessible surface area (SASA) of the chromophore and radial distribution of water molecules inside the β barrel. Sensors have different chromophores depending on the parental FPs: “CRO”, “CR2”, and “NRQ” are the three kinds of chromophores found in our selection, which are formed by three-residue sequences “TYG”, “GYG”, and “MYG”, respectively (Table 1 and Figure 1I). Along with the sensors, we analyzed their parental intact FPs: GFP, mRuby, and mNeonGreen bearing CRO, NRQ, and CR2, respectively. We investigate whether a higher Δ*F*/*F* correlates with a sensor structure where the opening created by circular permutation is occluded by the CaM-peptide domain to a greater degree. This is expected to lead to a similar environment for the chromophore to that of its parental FP. We further propose a mechanistic explanation of the regulation of the fluorescence state based on the shifts in occupancies of hydrogen bonds throughout the protein. We focus on GCaMP2 and jGCaMP8 as the two extremes of low- and high-performance sensors throughout the main text, while data on NCaMP7 and RCaMP1a are largely provided in the Supporting Information.

**Table 1:** Calcium Biosensors and Parental FPs Used in This Study

	**PDB code**	**glutamate with upshifted p*K*_a_**^a^	**p*K*_a_-calculated by Propka3**	**chromophore type**	**parental FP**	**Δ***F***/***F*
***GFP***	1EMA	E222	8.3	CRO	NA	NA
***mRuby***	3U0M	E215	4.4	NRQ	NA	NA
***mNeonGreen***	5LTR	E210	8.3	CR2	NA	NA
***GCaMP2***	3EVR	E135	7.4	CRO	GFP	4
***RCaMP1a***	3U0K	E133 (E97)	12.5	NRQ	mRuby	7.5
***NCaMP7***	6XW2	E45 (E35)	11.2	CR2	mNeonGreen	27
***jGCaMP8***	7ST4	E106 (E95)	7.8	CRO	GFP	75

a Index of glutamate residues in our MD inputs are given in parentheses in cases they are different from PDB files.

## 2 Methods

### 2.1 Modeling the Initial Coordinates of Intact FPs and *apo*/*holo* Sensors

We used ColabFold to complete the missing residues and to obtain an *apo*-like model for each sensor.^32^ Each *holo* sensor was modeled by providing the *holo* crystal structures given in Table 1 as homology templates. To obtain *apo* coordinates, we provided the two-calcium-bound GCaMP2 structure in the PDB-coded 3U0K as a template. However, we were able to obtain a relevant *apo* model only for GCaMP2, which we named apo*, while all other ColabFold predictions of *apo* forms resembled the *holo* structures, although these were not provided as template. *Apo*-like models were then obtained by manually removing the four calcium ions from the *holo* coordinates of the respective GEFBs. Three-residue sequences “TYG”, “GYG”, and “MYG” were used in place of CRO, CR2, and NRQ, respectively, as sequence inputs, because ColabFold does not recognize nonstandard residues.^33^

In the crystal structure of RCaMP1a, chromophore’s side chain has a slightly different chemical composition than its parental FP mRuby and is designated as “CRK”. To be able to compare the sensor to its parental FP, we have replaced the atom CRK-O1 in RCaMP1a with the structurally equivalent NRQ-N1 in mRuby (Figure 1I). In all systems, we used sequences without the purification tags and unresolved residues; therefore, residue indices are different in AF2 models (Figure S1).

The highest-ranked models in terms of prediction accuracy were selected and structurally aligned with their respective PDB templates, and the coordinates of the cyclized chromophores were transferred into the models. *Holo* models additionally contained four calcium atoms. All four sensors are in the high fluorescent state when calcium is bound. Therefore, we modeled all *holo* structures with an anionic chromophore and all *apo* structures with a neutral chromophore reflecting the ON and OFF states, respectively. The charge states of ionizable groups were predicted with Propka3,^34^ and all simulations were carried out with charge states at physiological pH. E222 of GFP, which is known to act as an acceptor in the light-induced proton transfer reaction, has an upshifted p*K*_a_.^35^ E95 of jGCaMP8, E35 of NCaMP7, E97 of RCaMP1a, E135 of GCaMP2, and E210 of mNeonGreen structurally align with E222, and all have upshifted p*K*_a_s and thus were modeled as neutral in the ON states (Table 1). Distinctively, structurally equivalent E215 of mRuby did not show such a p*K*_a_ upshift. It is important to note that we use the mRuby structure, which was crystallized at high pH to compare to RCaMP1a, since this is the pH range that the sensor is most efficient.^30^ All intact FPs were modeled with an anionic chromophore, reflecting the ON state (Table S1), along with neutral glutamates for GFP and mNeonGreen.

### 2.2 MD Simulations and Postprocessing

Simulation boxes were prepared with VMD and simulated with NAMD packages.^36,37^ Each protein was placed in a box of water with 150 mM KCl. At least a 10 Å layer of water in each direction from any atom in the system was added, so that there is at least 20 Å padding around the protein. All atoms were modeled using the CHARMM36 force field.^38^ The topologies of the neutral chromophores were used as described in CHARMM36 for residue labels “NRQ”, “CR2″, and “CRO”. Topologies of chromophores in the deprotonated form were derived by changing the charge distribution of atoms CE1, CE2, HE1, HE2, CZ, and OH based on the topology of the phenoxy group in CGenFF.^39^ The rest of the chromophore remained unchanged. Trajectories were calculated using the Verlet algorithm with a time step of 2 fs. The particle mesh Ewald method with a cutoff distance of 12 Å was used to calculate long-range electrostatics. Each system was subjected to minimization before running the NPT ensemble at 310 K and 1 atm for at least 400 ns (Table S1 and Figures S2 and S3). Coordinates were saved every 2 ps for trajectory analysis. Occupancies of hydrogen bonds involving the chromophore atoms were calculated using the VMD hydrogen bond plugin with a 3.5 Å donor–acceptor distance and 30° angle criteria. Occupancies of hydrogen bonds involving only standard residues were calculated with VMD Timeline plugin, and all hydrogen bonds between the atoms of two residues were merged into a single occurrence using previously published Python scripts.^40,41^ To select a homogeneous conformational population for further analysis, we carried out a cluster analysis using CPPTRAJ with the *k*-means algorithm based on C_α_ root-mean-square deviation (RMSD).^42^ Three conformational clusters were generated, and their fractions over the trajectory were plotted (Figure S2). Generated conformational populations are labeled as pop0, pop1, and pop2, with pop0 being the most abundant cluster observed in a run. CPPTRAJ analysis also provides a representative structure for each cluster as an output, which we use for visualization. All of the calculations were performed on the combined equilibrated trajectories of replicate runs (gray-shaded regions in Figure S2). *Holo-to-apo* morph movies were created with the ChimeraX “morph” command.^43^

Chromophore SASA was calculated using the Shrake-Rupley algorithm with a VMD Tcl script with a probe radius of 1.4 Å. Radial distribution function between water-oxygen atoms and the chromophore were calculated with VMD. Channels connecting the chromophore to the surface of the protein were calculated by the MOLE 2.5 web interface.^44^ The representative structures provided by the CPPTRAJ analysis for the dominant conformational cluster (pop0) were used for demonstrating the results of the channel calculations (Section 3.5). In addition, pop0 clusters of holo, apo and apo* states of GCaMP2 were analyzed to assess whether calculated channels reflect the conformational change around the cp site. The settings of MOLE 2.5 were selected with a bottleneck radius of 1.3 Å, bottleneck tolerance of 4 Å, and maximum tunnel similarity of 0.3, with the “Ignore HETATMs” option off to account for the space occupied by the chromophore. The Voronoi scale weight function was applied. Although the radius of the water molecule is 1.4 Å, we considered that a slightly smaller bottleneck radius would allow us to sample channels that might dynamically open up due to bottleneck fluctuations.^45^ Channels were visualized with ChimeraX. In some cases, channels with different sizes overlap; when this happens, only the larger channel is visualized.

Well-tempered metadynamics simulations were carried out on the calcium-removed *holo* structure of GCaMP2 with a bias temperature of 600 K, for a duration of 440 ns.^46^ The width and height of the Gaussian hills were set as 1.0 Å and 0.2 kcal/mol, respectively. Two collective variables (CVs) were defined, which reflect the *holo-to-apo* transition: The first CV is the distance between the CZ atom of the chromophore and the CZ atom of R377, the main hydrogen bond donor to the chromophore, and the second CV is the distance between the CZ atom of R81 on the FP domain and the CD atom of E387 on the CaM domain (see Section 3.1 for details on the choice of these CVs). The sample boundaries of both CVs are 2 and 20 Å.

## 3 Results

### 3.1 MD Equilibration and *holo*-to-*apo* Transition upon Calcium Removal

We observed different levels of conformational changes in response to calcium removal in the CaM domains among the four sensors studied. RMSD of the CaM-peptide domain between the dominant conformations of *apo* and *holo* are 3.9 Å for jGCaMP8, 2.3 Å for GCaMP2, 2.4 Å for RCaMP1a, and 3.4 Å for NCaMP7. RMSD plots show a greater backbone mobility for *apo* runs in all four sensors (Figure S2). The apo* run of GCaMP2, which started from the coordinates of two calcium-bound templates (PDB: 3O77), has even greater mobility (Figure S2D). In this structure, the two lobes of CaM are distant from each other, where the M13 peptide is closer to the N terminal lobe. The MD run showed a deviation from this structure; we observed that the two lobes approach each other, and the CaM domain becomes more compact (Movie S1). This conformation becomes dominant after nearly 30 ns (pop0 in the apo* plot in Figure S2D). All intact FPs showed considerably lower mobility and faster equilibration than the sensors in the ON state (Figure S3).

The CaM domain contains four EF-hand motifs. The change in the second EF-hand motif of jGCaMP8 (in sequential order) is displayed in Figure S4C. At this site, calcium is coordinated by one glutamate, three aspartates, one threonine, and one water. Upon removal of the calcium, coordinating residues pull away from each other due to electrostatic repulsion. A similar reorganization is observed in the other three calcium binding sites, although the exact content of binding residues differs while one water molecule is present in all (data not shown).

**Figure 2:**
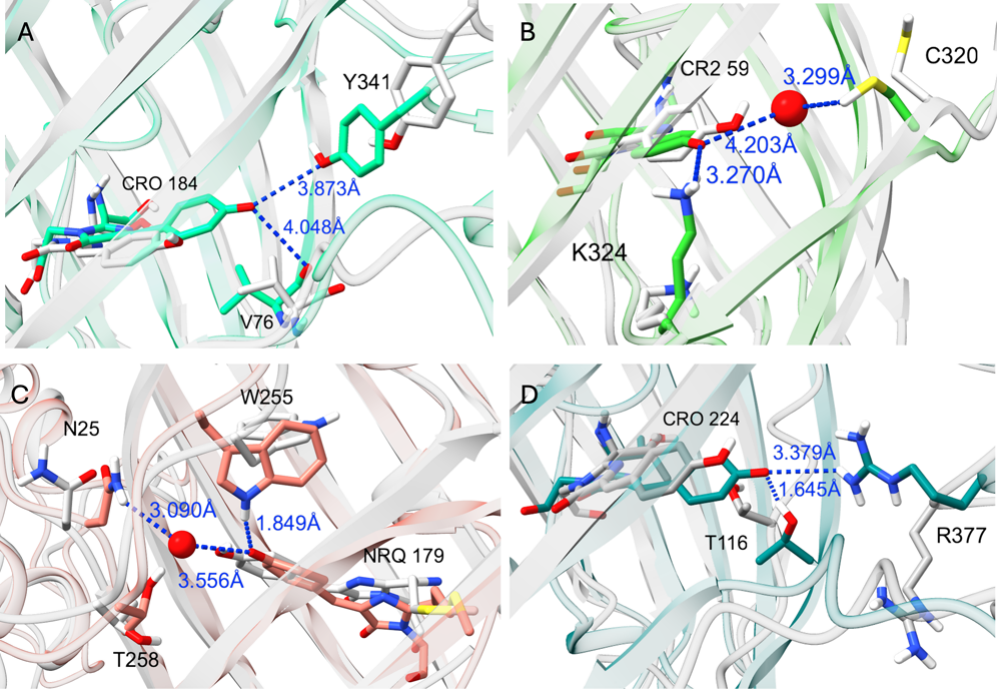
Hydrogen bond network near the chromophore in the ON and OFF states of each sensor. OFF states are colored gray, and ON states are colored according to the color emitted by the sensor. Images are taken from representative frames of each MD run and are structurally aligned with ChimeraX onto the FP domains. (A) jGCaMP8; (B) NCaMP7; (C) RCaMP1a; (D) GCaMP2. The hydrogen bonds between NRQ179-OH and N25-N and between CR2-OH and C320-SH are mediated by a water molecule. The water/oxygen bond is displayed as a red sphere. Distances are shown only for the *holo* states; only polar hydrogens of the side chains are explicitly shown.

**Figure 3:**
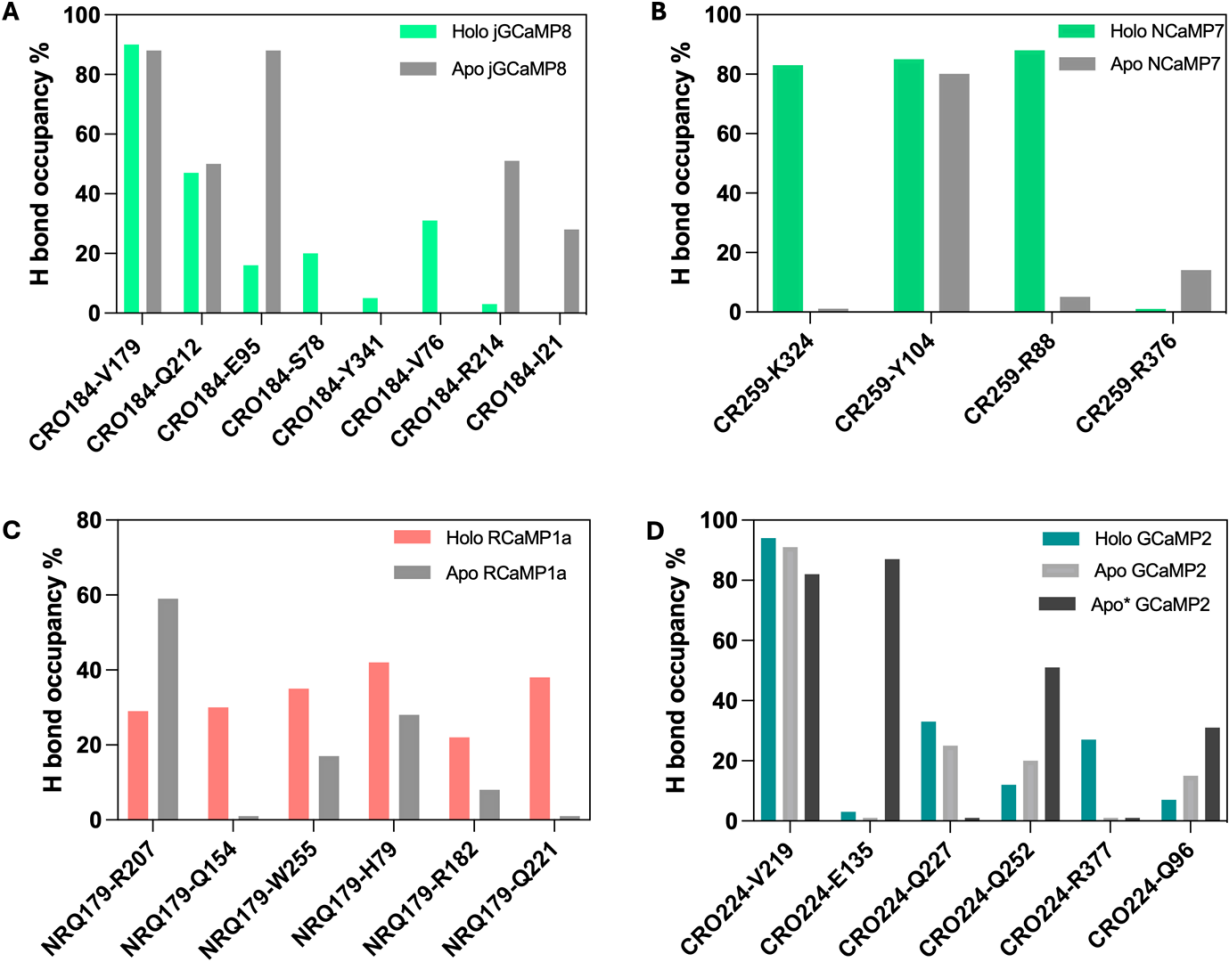
Occupancies of hydrogen bonds involving the chromophore as either an acceptor or a donor in *holo* and *apo* sensors. (A) jGCaMP8, (B) NCaMP7, (C) RCaMP1a, and (D) GCaMP2.

JGCaMP8 and GCaMP2 have similar positioning of cpGFP and CaM domains relative to each other, and calcium removal has a similar effect in both (Figure S4A,F). JGCaMP8’s CRO is hydrogen bonded to Y341 on helices 328–341, mostly through a water molecule, whereas GCaMP2’s CRO makes a salt bridge to R377 on the same helix (368–380 in 3EVR numbering) (Figure 2A,D). Upon calcium removal, this helix, along with the rest of the CaM domain, pulls away from the FP domain and is slightly distorted in both GCaMP2 and jGCaMP8 (Movies S2 and S3, respectively). Furthermore, we see a progressive change (gain or loss) of interaction with residues from within the β barrel going from the *holo* to fully *apo* state (Figure 3D). In both GCaMP2 and JGCaMP8, there is a significant reorganization of the chromophore lining residues within the β barrel in the *holo* to *apo* transition (Movies S2 and S3, respectively). This level of change in the positions of the β barrel residues is not observed in NCaMP7 and RCaMP1a (Movies S4 and S5, respectively).

Although RCaMP1a has the same circular permutation strategy as GCaMP2 and JGCaMP8, the position of the M13 peptide relative to FP in the three-dimensional structure is quite different (Figure 1C). The crystal structure reveals T258 as the only hydrogen bond donor to the phenoxy oxygen of the chromophore. Our results indicate that W255 on the FP and N25 on the linker also take part in stabilizing the anionic chromophore (Figure 2C and Figure 3C), although interactions with N25 is mediated by water molecules.

In NCaMP7, the intact CaM-M13 sequence is inserted into mNeonGreen (Figure 1H). Because of the covalent connection to the CaM domain, the M13 helix moves along with the CaM domain and changes its position relative to the FP to a greater degree than the other three sensors (Figure S4D). There is a clear shift in the position of hydrogen-bonded residue pairs at the cp site, as well as the hydrogen bond donors of the anionic chromophore (Movie S4). The NCaMP7 chromophore has two hydrogen bond donors on FP, K324, and one gate post residue C320 (Figure 2B). The high occupancy of K324-NRQ interaction in the *holo* state (80%) relative to *apo* is probably due to the negative charge on the chromophore rather than a conformational difference (Figure 3B). The hydrogen bond with C320 is mediated by a water molecule. The distance between the chromophore phenoxy oxygen and its primary hydrogen bond donor increases when going from *holo* to *apo* state in all three sensors, except for NCaMP7 (Figure S5). The CR2(OH)-C320(SH) distance in the *apo* state still allows for a water-mediated hydrogen bond (Figure S5B).

**Figure 4:**
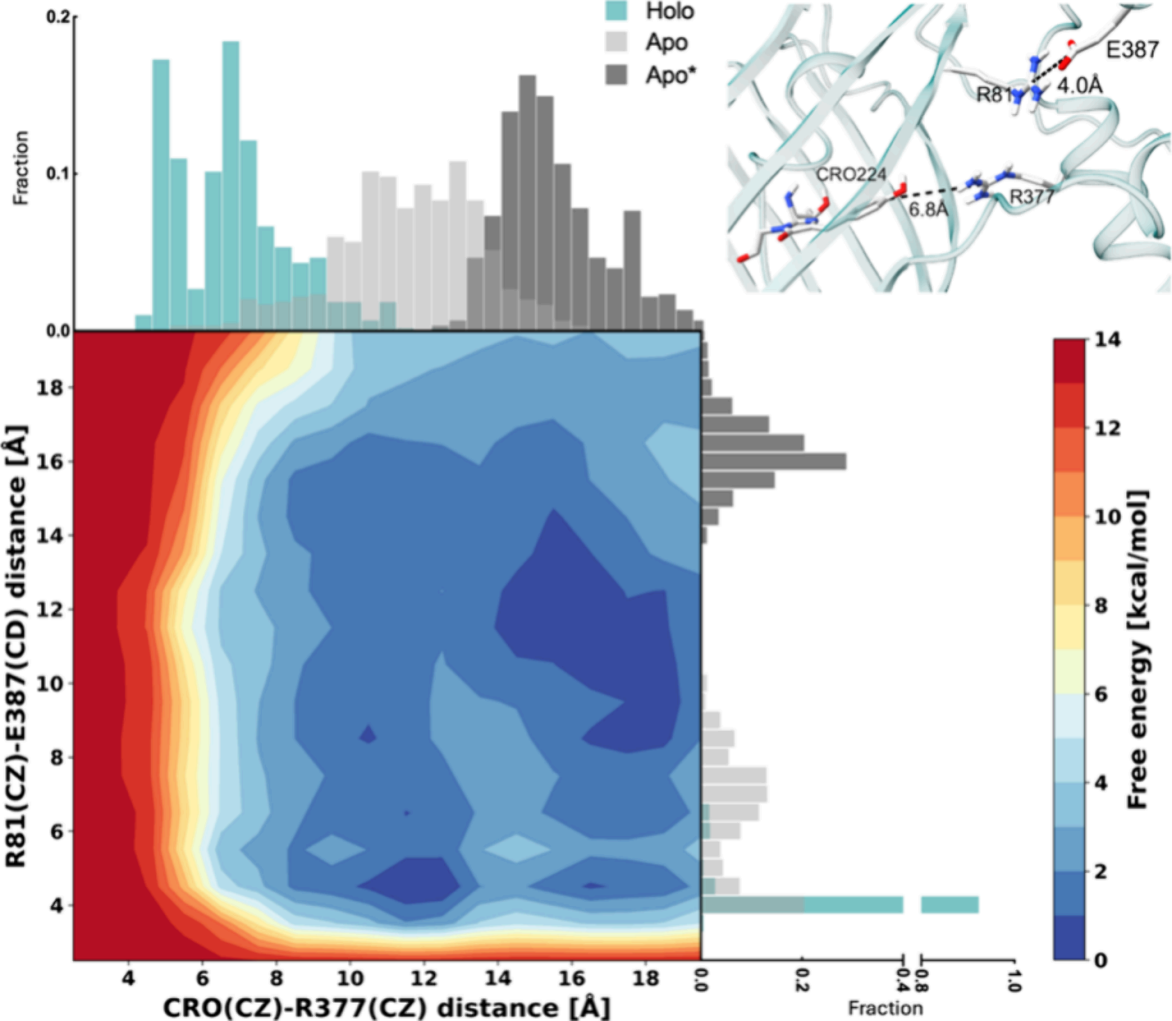
Potential of mean force calculated via well-tempered metadynamics simulations on calcium-removed *holo* GCaMP2. The frequency distribution of the distances of the selected CVs in *holo* (green), *apo* (light gray), and apo* (dark gray) states are shown along the axes. The positions of the selected distances along the interface relative to the chromophore are shown on the upper right.

To see if triggering the *apo* forms simply by removing the ligands from the *holo* structures serve our purposes and sets the system on a pathway to equilibrated *apo* structures, we performed well-tempered metadynamics simulations on GCaMP2 (started from *holo* GCaMP2 with Ca^2+^ ions removed). We chose GCaMP2 to carry out the metadynamics simulations since it has an *apo*-like crystal structure, which we termed apo*, that we can utilize to judge whether the initial structure eventually samples a similar structure under the applied bias potential. The axes along the sides in Figure 4 shows the distribution of critical distances at the interface of the *holo* structure through the classical MD simulations, for *holo, apo*, and apo* states (Figure S2D). Note that the hydrogen bonds are calculated between the donor–acceptor pairs, while here we display the values between the nearest carbon atoms. As we observed for all four sensors, the hydrogen bond donor to the chromophore phenoxy oxygen pulls away in the classical MD simulations, albeit to varying degrees, in the *apo* state (Figure S5). This distance gets even larger in the apo* state of GCaMP2, therefore presenting itself as an obvious choice as a CV. We selected the second CV as R81-E387 distance since we observed a significant loss in the hydrogen bond occupancy between these two interface residues during the course of *holo-to-apo* transition in classical MD simulations (a drop from 96 to 7% occupancy) (Table S2). Similar to the loss of this hydrogen bond, the distance distribution between the two residues becomes significantly wider in the *apo* state. Using these two CVs as relevant to driving the *holo-to-apo* transition, the calculated free energy landscape shows that both CVs readily sample the regions covered by the classical MD simulations of *apo* and apo* systems (Figure 4). This result shows that removing the ligand from the initial *holo* structure triggers a structural change toward the *apo* form from which the hydrogen bond loss/gain calculations described in the next subsection were carried out, and validates our standard MD-based approach for the other three sensors.

### 3.2 Hydrogen Bond Networks Distinguish the Dark vs Bright States

**Figure 5:**
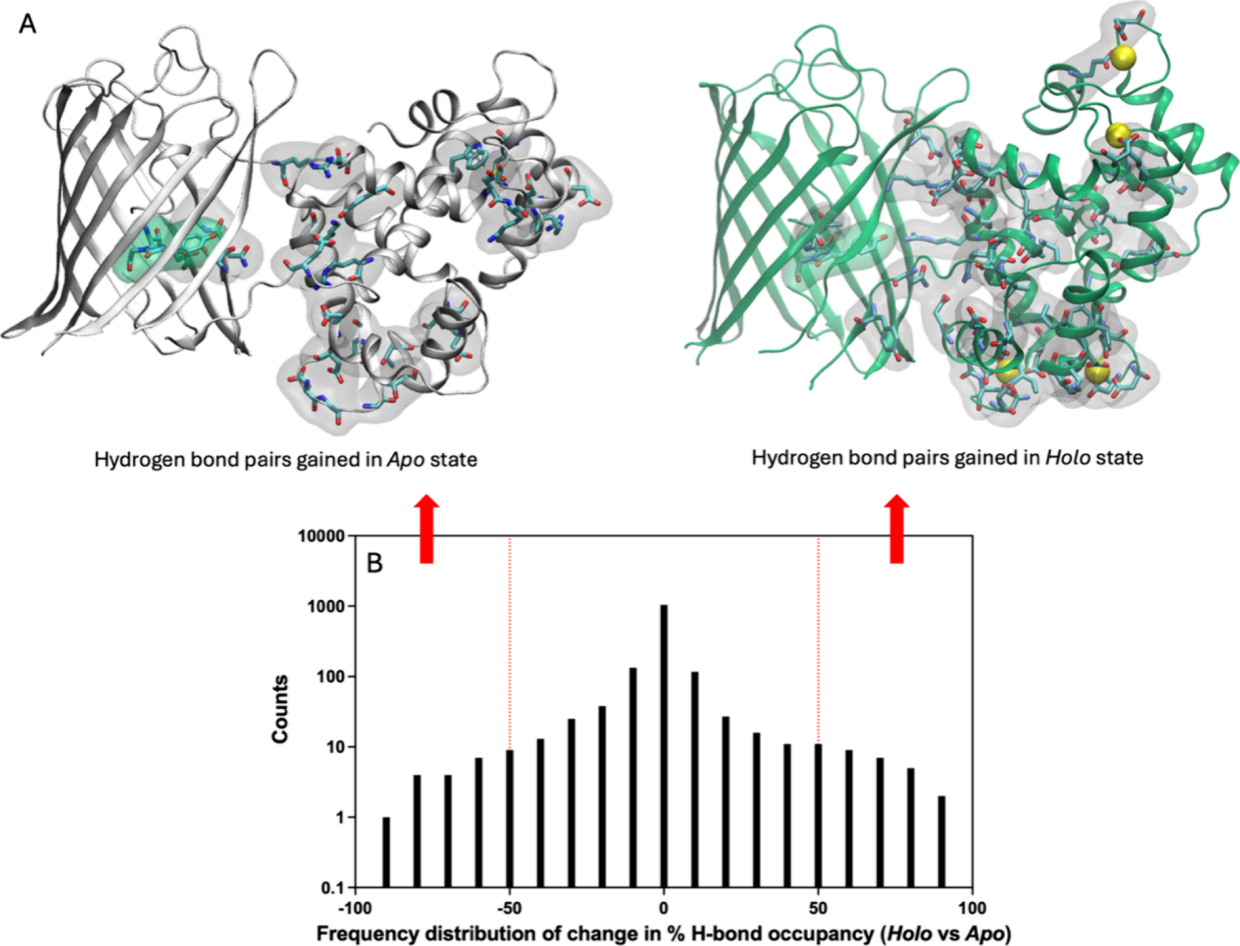
Shifts in hydrogen bond occupancies between *holo* and *apo* states of GCaMP2 differentiate the ON/OFF states. (A) Hydrogen-bonded residue pairs whose occupancy increases (decreases) by more than (less than) 50% in *holo* (*apo*) states visualized as licorice and surface representations. (B) Histogram of difference in % hydrogen bond occupancies in *holo* vs *apo* states; arrows indicate the hydrogen bond selected to be plotted in (A). For clarity, *y*-axes are plotted on a logarithmic scale.

To reveal the allosteric effect of calcium removal on the chromophore environment, we analyzed the shifts in hydrogen bond occupancies over the whole structure. We listed the residue pairs with altered hydrogen bond occupancies beyond a certain threshold to discern hydrogen-bonded residue pairs that show significant deviations between the *apo* and *holo* forms. We excluded shifts in occupancies with pairs of residues located on FP outer loops, since they are highly flexible and probably not related to the *holo-to-apo* conformational transition. We determined the threshold for each system based on the distribution of occupancy differences between *holo* and *apo*. Similar to our previous work on a maltose biosensor, the majority of hydrogen bond occupancies remain within ±50% change interval when the *apo* and *holo* runs are compared.^14^ We found that the shift in hydrogen bond occupancies characterized the conformational change in the CaM domain that is communicated to the chromophore environment. In fact, a distinctive feature of ON state sensors is a continuous network of hydrogen bonds from the calcium binding site to the chromophore environment. Figure 5A shows the location of hydrogen-bonded residue pairs in GCaMP2 whose occupancies change by more than 50% when going from the *holo* to *apo* states. The *holo* state has considerably more hydrogen-bonded pairs at calcium binding sites as well as at the interface between the FP and the CaM domain. These interactions are progressively lost when going from *holo* to *apo* to apo* states (compare Figure 5 to Figure S6D and see also Table S2) with a higher number of hydrogen bonds completely lost or gained along the way. Still, manual removal of calcium ions and the initiation of the *apo*-like structure provides sufficient insight for changes occurring in the hydrogen bond distributions, especially of residue pairs at the FP-CaM interface that are the main cause of the loss of fluorescence, not only for GCaMP2 but for all the sensors studied in this work (Figure S6A–C). As a general feature, the accumulated hydrogen bonds display a continuous network in ON states, while they are fragmented in OFF states in all of the sensors studied. However, one must be cautious as the difference in hydrogen bond occupancies observed between *holo* and *apo* states would depend on how well the *apo* state is sampled starting from the *holo* coordinates. For example, in the case of RCaMP1a, some interactions around the linker area linger in the *apo* state (Figure S6C).

### 3.3 Solvent Accessibility of the Chromophore Is Not a Direct Indicator of Fluorescence Efficiency

**Figure 6:**
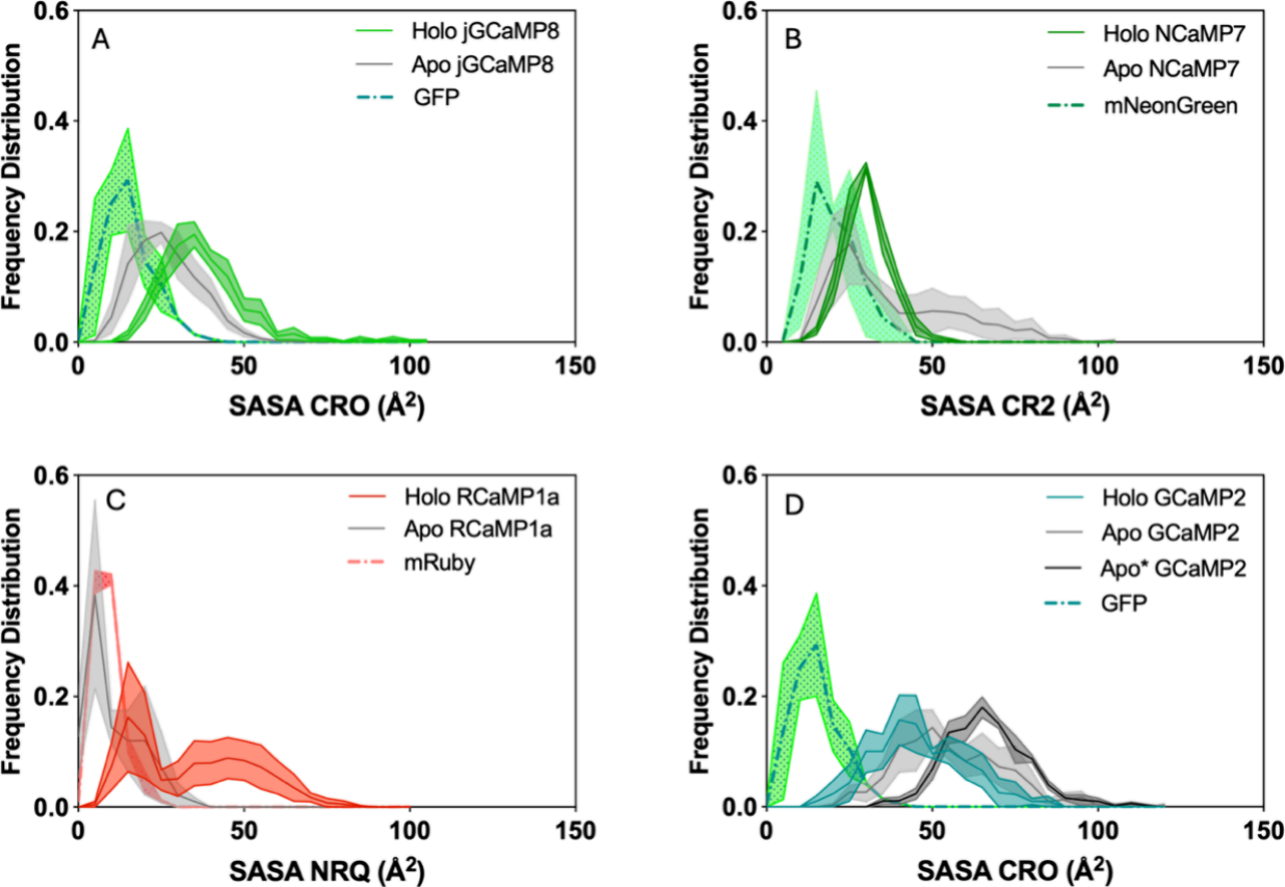
Frequency distribution of chromophore SASA grouped according to chromophore type. (A) jGCaMP8 and GFP. (B) NCaMP7 and mNeonGreen. (C) RCaMP1a and mRuby. (D) GCaMP2 and GFP.

We grouped the frequency distribution of SASA values for each chromophore type. We hypothesized that a small difference in chromophore SASAs between the sensor and its parental FP may indicate that the opening created by circular permutation is occluded well, and there is less solvent access into the β barrel, which may promote the high fluorescent state. The ON state chromophores of all four sensors have higher SASA values than their parental FPs (Figure 6). Furthermore, NCaMP7 and GCaMP2 sensors showed an increase in average SASA in the *apo* state chromophore compared to *holo* state chromophores (Figure 6B,D). In GCaMP2 apo*, this increase is more pronounced. In contrast, *holo* state chromophores of JGCaMP8 and RCaMP1a reached higher SASA values than *apo* states (Figure 6A,C). RCaMP1a’s, *apo* state chromophore shows SASA values even lower than the intact mRuby (Figure 6C). This discrepancy can be attributed to the change in the surface area of chromophore itself in the anionic vs neutral state and the reorganization of β barrel residues lining the chromophore. We argue that the SASA change created by opening at the cp site as well as by the loss of contact with hydrogen bond donors as a result of *holo*-to-*apo* transition can be compensated by the changing positions of residues lining the chromophore. This effect may be especially pronounced in jGCaMP8, where a bulky tyrosine residue pulls away from the chromophore, creating a larger opening at this site. However, there is also a significant reorganization of chromophore lining residues within the β barrel in this sensor during the *holo-to-apo* transition, which may offset the expected increase in chromophore SASA (Movie S3). This result indicates that the change in chromophore SASA is not a good indicator to judge the conformational changes occurring around the cp site due to the *holo-to-apo* transition. In fact, we find that the compensatory effects call for an analysis of noncontact interactions. We therefore decided to measure the actual distribution of water molecules surrounding the chromophore, as we explain in the next section.

### 3.4 Water Number Density Near the Chromophore Is a Direct Indicator of Fluorescence Efficiency

**Figure 7:**
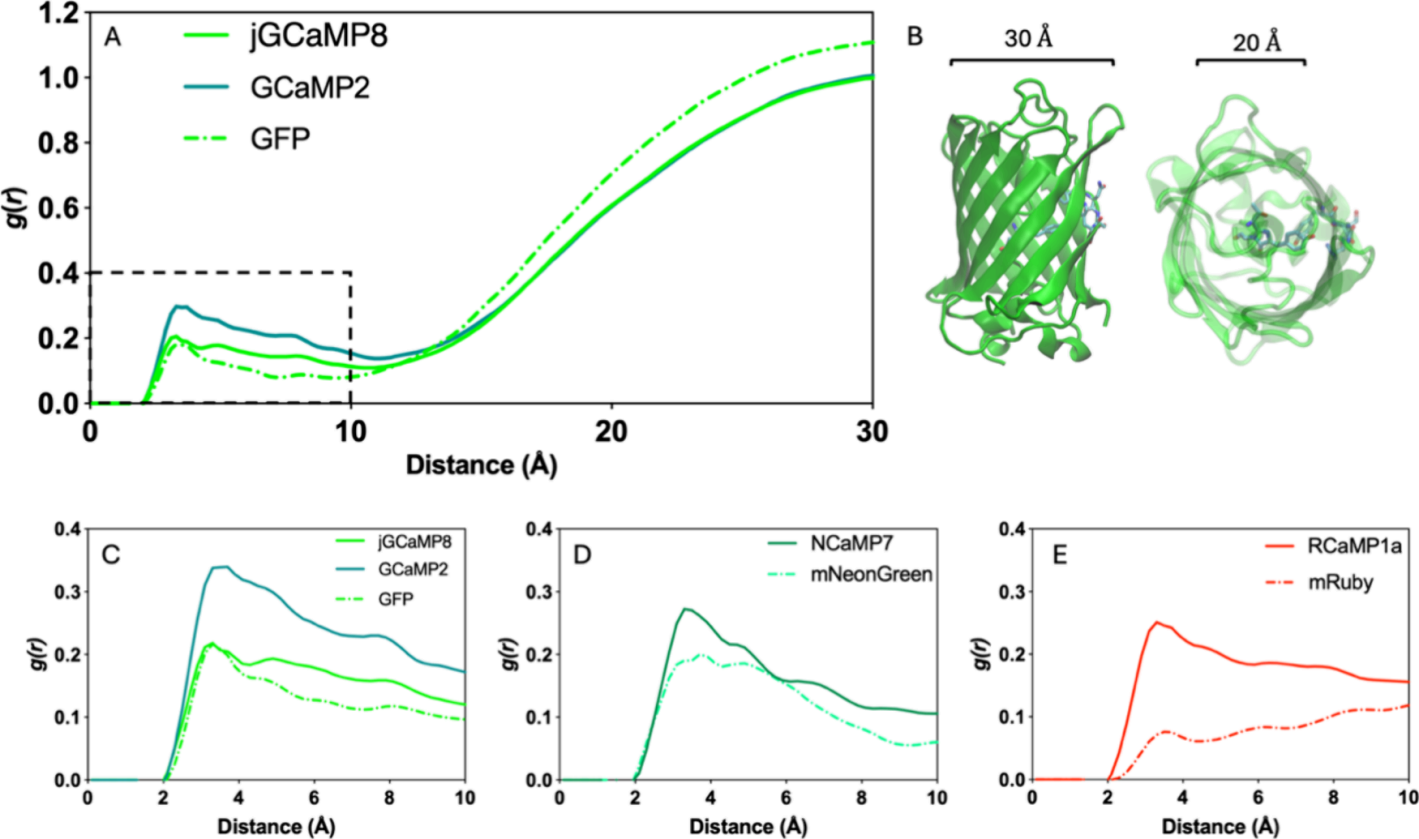
Radial distribution function (RDF) of water-oxygen around the chromophore in *holo* sensors and intact FPs. (A) RDF of water-oxygen up to 30 Å from the chromophore in GFP and GFP-derived sensors. (B) Dimensions of GFP. (C–E) RDF of water-oxygen up to 10 Å in *holo* sensors and their ON state parental FPs (dashed).

As a measure of openness at the cp site, we examined the distribution of water molecules in the vicinity of chromophore (Figure 7). Radial distribution function (RDF; *g*(*r*)) describes the density of a chosen group of molecules around a reference point as a function of distance between them and tends to 1 at distances far from the central atoms (Figure 7A). We study this property based on the fact that the efficiency and dynamics of excited-state proton transfer in fluorescent proteins are governed by an intricate interplay of chromophore structure, hydrogen bonding networks, electrostatic interactions, protein conformation, pH conditions, and quantum mechanical effects.^3,47−50^ The accessibility of the chromophore as a whole is the deciding factor for the amount of quenching that will occur in the individual sensor since there are many compensating effects in the local environment that are crucial for the proton transfer, as exemplified in the previous subsection. The essential role of water molecules in the reaction is multifaceted and includes functioning as mediators in proton transfer, stabilizing transition states, enhancing fluorescence properties, and supporting the structural changes needed for efficient reactions. Their presence and positioning within the protein structure are vital for the optimal fluorescence performance and stability. We therefore select the total water in the proximity of the chromophore by measuring the radial distribution of water molecules within the 10 Å distance, which corresponds to the inner radius of the β barrel of the fluorescent domain (Figure 7B).

RDF plots show a significantly reduced number of water molecules around the chromophore in all three parental FPs compared with the sensors (Figure 7C–E). This is a plausible result since the chromophore rests within the undisrupted β barrel in intact FPs. For all systems, a common peak at around 3.3 Å points to the first layer of solvation. We found that the Δ*F*/*F* of the sensors are correlated with the ratio of numerical integral of the *g*(*r*) curve to that of its parental FP. Ratios of integrated area up to 10 Å are JGCaMP8/GFP = 1.18, NCaMP7/mNeonGreen = 1.32, RCaMP1a/mRuby = 1.56, and GCaMP2/GFP = 1.83. This ordering shows that the smaller the ratio, the larger the Δ*F*/*F* of the four sensors listed in Table 1 and points to a distinctive feature between sensors of low and high Δ*F*/*F*: High Δ*F*/*F* sensors effectively allow a smaller number of water molecules around the chromophore when normalized by the values in their respective intact FPs. Structurally, we can argue that the opening created near the cp site is occluded by the SD to a greater extent in sensors of high Δ*F*/*F*.

We note that we also calculated the RDFs between individual atoms of the chromophore and water. It turns out these calculations prove less informative due to their rather jagged features since we have only a single atom to use as a center; this contrasts with all chromophore heavy atoms used for constructing Figure 7 (a sample calculation for the N2 atom is provided in Figure S10). Nevertheless, these individual plots also contribute to our main conclusion drawn from the RDF curves, that the sensors generally have more water penetration toward their chromophores than their intact fluorescent protein counterparts. Moreover, the smaller the difference, the higher the Δ*F*/*F* value, although it is harder to quantify the differences for the individual atom analyses since the local environment is different for each case and the charge states of the residues that are in direct contact with the chromophore might also differ depending on the p*K*_a_ calculations (Table 1), which ultimately affect the RDFs based on individual chromophore atoms. Thus, the overall behavior for the chromophore proves to be the most informative, as the dynamics compensate for these various effects to shape the final chromophore environment.

### 3.5 The More Organized Solvent Channels around the cp Site Lead to More Efficient Fluorescence

As a qualitative evaluation of the openness around the cp site, we visualized the solvent channels connecting the chromophore to the surface using MOLE 2.5 software. Channels are described as passages connecting a point inside the protein to the surface, i.e., they detect if a probe of specified size may find a path to the interior.^45^ Channels have been studied, both by crystallography and by MD simulations, to reveal substrate/product transport pathways in enzymes^51,52^ or the water passage in aquaporins.^53^ ChannelsDB 2.0 offers a comprehensive resource of different examples of channels and pores in biomacromolecules.^54^ In the case of fluorescent sensors, we consider near water-sized channels around the cp site as passages for water to access the inside of the β barrel.

**Figure 8:**
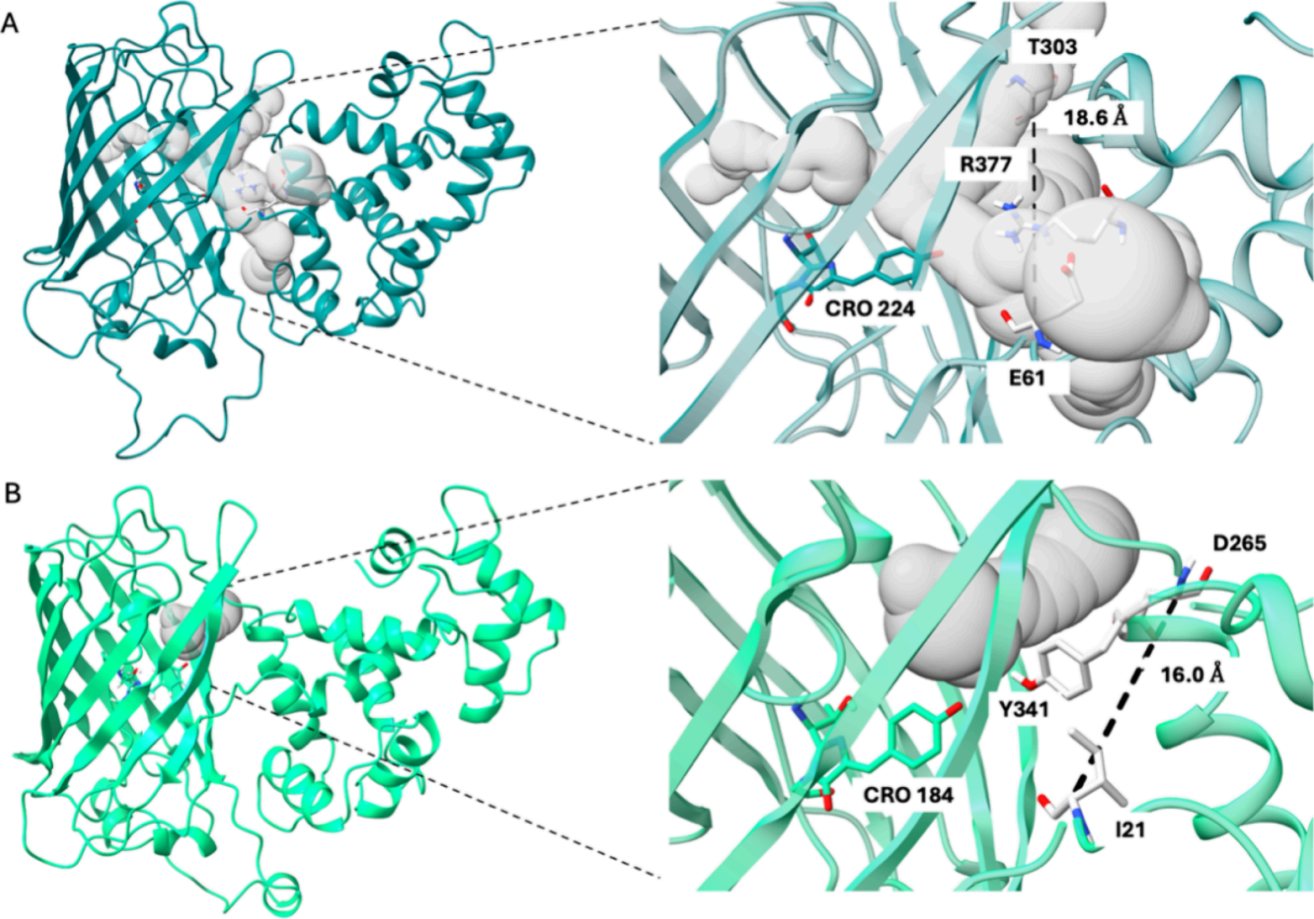
Channels connecting the surface to the chromophore. (A) *holo* GCaMP2 and (B) *holo* jGCAMP8. Insets show close-up of channels and distances between gate post residues (T303 and E61 in GCaMP2, I21 and D265 in jGCaMP8) flanking the bulge.

When comparing the *holo* states of GCaMP2 (having the smallest Δ*F*/*F* in our set) and jGCaMP8 (having the largest Δ*F*/*F* in our set), more voluminous channels are found near the GCaMP2 chromophore (Figure 8). These channels surround the residues between the chromophore, gate post residues, and residues from the CaM domain interacting with the FP domain. In jGCaMP8, the bulky tyrosine residue (Y341) from the CaM domain seems to fill up this space more effectively. Additionally, the side chain of gate post residue I21 also faces inward, while both gate post residues of GCaMP2 face toward the solvent. We also found that the distance between C_α_ atoms of two gate post residues are the greatest in the case of GCaMP2 (Figure 8 and Figure S7), even though it has a very similar overall structure to jGCaMP8 (C_α_ RMSD of 0.3 Å between their *holo* states). A larger distance between the gate post residues also increases the likelihood of finding a channel around the cp site. Furthermore, we found that the total volume of the channels around the cp site increases going from the *holo* to *apo* state in GCaMP2 (Figure S8).

Visualizing solvent channels around the cp site provides a qualitative way to evaluate the degree of chromophore exposure from the experimental or predicted PDB structures. However, it may be limited to differentiating sensors with very high Δ*F*/*F* difference, as in the case for GCaMP2 and jGCaMP8. In NCaMP7, channels extend between the two gate post residues as in GCaMP2 and JGCaMP8, whereas in RCaMP1a, we detected a different channel opening up in between the adjacent two strands in one of the duplicate runs (Figure S9). This opening is not observed in the initial structure of RCaMP1a, although a slight distortion is observed in the strand adjacent to the cp site (strand 4 covering residues 111 to 122 in PDB: 3U0K). Although the distance between gate post residues is significantly lower in RCaMP1a than other three sensors, water may access into the β barrel through this alternative opening.

We emphasize that due to the dynamic nature of these channels, it is not possible to use them as quantitative measures of chromophore accessibility; moreover, there is no reliable way to report their volumes due to the open-ended nature of the channels. However, they assess qualitative features; i.e., the channels of GCaMP2 are larger and more in number, while those of jGCaMP8 are fewer and display smaller volumes.

## 4 Discussion

In this work, we used classical MD simulations to shed light on the working mechanism of a series of CaM-based single FP biosensors with varying degrees of Δ*F*/*F.* We show that the dynamic arrangement within at least 10 Å of the chromophore must be included for a good model of these GECIs. However, the arrangement of the atoms in the complex biomolecular environment significantly increases the number of atoms and interactions, making calculations at a quantum mechanical (QM) level impractical for a comprehensive analysis. Also, truncated QM models may fail to capture the full influence of the environment of the chromophore. On the other hand, simplifying the model for feasibility would compromise the reliability of any conclusions drawn regarding the change in fluorescence. In addition, QM calculations, particularly those involving excited states needed for fluorescence analysis, have extra computational demands. With the currently available computers, QM treatment might allow for studying a single system, which is at odds with our aim to lay out a computationally tractable pipeline that allows for comparing several systems and providing a prediction of their relative performance.

In fact, our MD simulations provided intriguing insights into the relation between biosensor dynamics and calcium binding-dependent fluorescence change. One key observation was that sensors with enhanced Δ*F*/*F* values have a limited distribution of water molecules around the chromophore. Water exposure is known to cause protonation of the phenoxy group of chromophore and quenching of the fluorescence.^55^ Apart from protonation, water can also alter the chromophore local environment and disrupt the hydrogen bond network within the β barrel.^56^

It is known that the fluorescence emission of GFP heavily depends on the hydrogen bond network and the surrounding water molecules.^5,57^ We found that intact FPs without circular permutation have considerably less water around the chromophore. Our analysis revealed a significant difference between the two extremes of our selection: GCaMP2 (Δ*F*/*F* = 4) and jGCaMP8 (Δ*F*/*F* = 75). The two sensors share a high level of structural homology, despite the differences in CaM-binding peptide sequences and a few mutations at the interface between FP and CaM. We further probed solvent channels around the cp sites. In agreement with the higher number of water molecules within the β barrel of GCaMP2 compared to jGCaMP8, more pronounced solvent channels that extend between two gate post residues are detected. These channels may also reflect how well the CaM and FP domains pack against each other; a higher degree of packing allows for less solvent access to this area. RCaMP1a, with the smallest gate post distances, actually has a smaller opening at the cp site than the other three sensors; however, we found that the adjacent strand has a propensity to distort and allow for solvent access to the interior.

It should also be noted that the ligand binding-dependent fluorescence increase may be due to different factors in different sensors. For example, the mechanism of fluorescence response of RCaMP1a is based on changes in the fluorescence lifetime and quantum yield rather than a shift in the relative populations of anionic vs neutral chromophores,^30^ as is the case for GCaMP2 where the anionic chromophore is stabilized by R377 in the CaM domain.^13^ This shift is reflected in the ability of the chromophore to absorb light. On the contrary, for jGCaMP8 and NCaMP7, the most significant contribution to increased fluorescence efficiency is the increase in quantum yield upon calcium binding.^28,29^ Although the working principle of all CaM-based sensors is based on the calcium binding induced conformational change, their fluorescence responses are fine-tuned by extensive protein engineering, such as rigidifying the chromophore environment to suppress the nonradiative decay pathways^58^ or any structural optimization that may stabilize the excited state, extending the fluorescence lifetime. Water access to the vicinity of the chromophore, which has been the focus of this study, may affect all of these processes. Water can decrease the quantum yield by collisional quenching^48,50^ or change the electronic structure of the chromophore, by stabilizing the ground and excited states differently.^47,49^ In fact, our results show that the distribution of water inside the β barrel is a defining parameter for the efficiency of these sensors despite the differences in physical mechanisms affecting their fluorescence response. However, a full exploration of how water affects each of these parameters, which may require quantum chemical calculations, is beyond the scope of this paper. Nevertheless, future QM/MD studies may address this issue, and the modeling approach used therein might rely on some of the insights provided in this work.

As a second indicator of sensor efficiency, we evaluated the change in chromophore SASA of the sensors in ON/OFF states compared to those in ON state parental FPs. SASA of the chromophore depends on residues lining its vicinity, including some residues at the linker area and within the β barrel. Our results showed that, even though residues that act as hydrogen bond donors to the phenyl oxygen of the chromophore move away and create a larger opening at the cp site, this change does not always translate into an increase in the chromophore SASA. We argue that due to the highly buried state of the chromophore within the barrel, any change in the chromophore’s shape, such as the coplanarity of the two rings, as well as the respositioning of chromophore lining residues may affect the measured SASA values.

Our analysis of hydrogen bond occupancies between ligand-bound and ligand-free sensor structures provided additional mechanistic insights into the allosteric modulation of the chromophore environment. Shifts in hydrogen bond occupancies have been shown to regulate the functioning of other proteins as well.^40,59,60^ Our findings suggest that the shifts in hydrogen bond occupancy patterns upon calcium binding serve as a means to transmit conformational changes occurring in the CaM domain to the chromophore environment. While the vast majority of the hydrogen bond occupancies remains within ±50% in the *holo* and *apo* runs, those that change beyond ±50% provide a clue on how the conformational change affects the whole structure. Notably, a distinct characteristic of the ON state sensors is the presence of a continuous network of hydrogen bonds extending from the calcium binding site to the chromophore environment. This is a common profile observed in all four sensors we study in this work, irrespective of the value of Δ*F*/*F,* and appears to be a requirement for the chromophore to function. In future studies, this hypothesis could be further tested by modeling failed sensor designs reported in the literature. We note that, in particular, the hydrogen bond network is disrupted near the FP-SD interface in the *apo* state. We consider this parameter an important structural checkpoint when assessing the sensor performance. Furthermore, we find that the hydrogen bonds disrupted in this region also provide reliable reaction coordinates for enhanced sampling of the conformations; this insight provides physics-based CVs that are crucial in navigating the conformational space of proteins. In conclusion, our results form the basis for understanding the structural determinants of high performance biosensors and suggest that MD simulations can provide guidance in the optimization of insertion sites and linker residues to aid in the reduction of the experimental screening time and costs.

## Data Availability

All raw data and analysis scripts are deposited at 10.5281/zenodo.14199597

## Supporting Information Available

Clustal Omega multiple sequence alignment of the four sensors; list of MD simulation runs; backbone RMSD and conformational cluster fraction plots; changing positions of CaM and FP domains relative to each other as a result of calcium removal from the*holo* sensor; distribution of interatomic distances between chromophore phenoxy oxygen and its primary hydrogen bond donor in the *holo* (ON) and *apo* (OFF) states; shift in hydrogen bond occupancies between *holo* and *apo* sensors; progressive change of hydrogen bond occupancies in going from *holo* to *apo** state in GCaMP2; interatomic distances between C_α_ atoms of gate post residues in the *holo* state of each sensor; close up and overall top-down views of representative solvent channels in GCaMP2; solvent channels in *holo* RCaMP1 and NCaMP7; and radial distribution function of water–oxygen within 10 Å of the chromophore N2 atom (PDF)
Apo* GCaMP2 trajectory (MP4)
*holo-to-apo* transition in GCaMP2 (MP4)
*holo-to-apo* transition in JGCaMP8 (MP4)
*holo-to-apo* transition in RCaMP1a (MP4)
*holo-to-apo* transition in NCaMP7 (MP4)

## Abbreviations

cp: circular permutation
GFP: green fluorescent protein
CaM: calmodulin
SD: sensory domain
MD: molecular dynamics
RDF: radial distribution function
RMSD: root-mean-square deviation.
