# Supplementary material for: Ranking Single Fluorescent Protein-Based Calcium Biosensor Performance by Molecular Dynamics Simulations
Source: J Chem Inf Model. 2024 Dec 27;65(1):338–50. doi: 10.1021/acs.jcim.4c01478 (PMC11733952; doi:10.1021/acs.jcim.4c01478)
Supplement: Supplementary file 1 — ci4c01478_si_001.pdf [file ci4c01478_si_001.pdf]

Supplementary material for

Ranking Single Fluorescent Protein Based Calcium Biosensor Performance by Molecular Dynamics Simulations

Melike Berksoz, Canan Atilgan*

Faculty of Engineering and Natural Sciences, Sabanci University

***Correspondence:** Canan Atilgan, Faculty of Natural Sciences and Engineering, Sabanci University, Tuzla 34956 Istanbul, Turkey, E-mail: canan@sabanciuniv.edu

```
NCaMP7     MGGSHHHHHHGMASMTGGQQMGRDLYDDDDKEN--------------------------- 33
RCaMP1a    -MGSHHHHHHGMASMTGGQQMGRDLYDDDDKDLATMVDSSRRKWNKTGHAVRAIGRLSSA 59
jGCaMP8    ------------------------------MHHHHHHTRRKKTFKEVANAVKISASLM-G 29
GCaMP2     -------------------------------------DSSRRKWNKTGHAVRAIG-RL-S 21

NCaMP7     ------------------------------------------------------------ 33
RCaMP1a    INTEMMYP---ADGGLRGYTHMALKVDGGG-HLSCSFVTTYRSKKTVGNIKMPGIHYVSH 115
jGCaMP8    LKINVYIKADKQKNGIKANFHIRHNIEDGGVQLAYHY--QQNTPIGDGPVLLPDNHYLSV 87
GCaMP2     SLENVYIMADKQKNGIKANFKIRHNIEDGGVQLAYHY--QQNTPIGDGPVLLPDNHYLST 79

NCaMP7     --------------------------------LYFQGHMRSMVSKGEEENMASLPATHEL 61
RCaMP1a    RLERLEES---DNEMFVVQREHAVAKFVGL--GGGGGTGGSMNS----LIKENMR--MKV 164
jGCaMP8    QSKLSKDPNEKRDHMVLLEFVTAAGITLGMDELYKGGTGGSMVSKGEELFTGVVP--ILV 145
GCaMP2     QSKLSKDPNEKRDHMVLLEFVTAAGITLGMDELYKGGTGGSMVSKGEELFTGVVP--ILV 137

NCaMP7     HIFGSINGIDFDMVGQGTGNPNDGYEELNLKS-TMGDLQFSPWILVPHIGYGFHQYLPYP 120
RCaMP1a    VLEGSVNGHQFKCTGEGEGNPYMGTQTMRIKVIEGGPLPFAFDILATSFMYGSRTFIKYP 224
jGCaMP8    ELDGDVNGHKFSVSGEGEGDATYGKLTLKFI-CTTGKLPVPWPTLVTTLTYGVQCFSRYP 204
GCaMP2     ELDGDVNGHKFSVSGEGEGDATYGKLTLKFI-CTTGKLPVPWPTLVTTLTYGVQCFSRYP 196

NCaMP7     DGMSPFQA-AMVDGSGYQVHRTMQFEDGASLTVNYRYTYEGSHIKGEAQVEGTGFPADGP 179
RCaMP1a    KGI--PDFFKQSFPEGFTWERVTRYEDGGVITVMQDTSLEDGCLVYHAQVRGVNFPSNGA 282
jGCaMP8    DHMKQHDFFKSAMPEGYIQERTIFFKDDGNYKTRAEVKFEGDTLVNRIELKGIDFKEDGN 264
GCaMP2     DHMKQHDFFKSAMPEGYIQERTIFFKDDGNYKTRAEVKFEGDTLVNRIELKGIDFKEDGN 256

NCaMP7     VMTNSLTA--EAHDQLTEEQIAEFKEAFSLFDKDGDGTITTKELGTVMRSLGQNPTEAEL 237
RCaMP1a    VMQKKTKGWEPTRDQLTEEQIAEFKEAFSLFDKDGDGTITTKELGTVMRSLGQNPTEAEL 342
jGCaMP8    ILGHKLEY--NLPDQLTEEQIAEFKEAFSLFDKDGDGTITTKELGTVMRSLGQNPTEAEL 322
GCaMP2     ILGHKLEY--NTRDQLTEEQIAEFKEAFSLFDKDGDGTITTKELGTVMRSLGQNPTEAEL 314

NCaMP7     RVMIIEVDADGDGTLDFPEFLAMMARKMKYRDTEEEIREAFGVFDKDGNGYIGAAELRHV 297
RCaMP1a    QDMINEVDADGDGTIDFPEFLIMMARKMKDTDSEEEIREAFRVFDKDGNGYISAAELRHV 402
jGCaMP8    QDMINEVDADGDGTIDFPEFLTMMARKMKYRDTEEEIREAFGVFDKDGNGYISAAELRHV 382
GCaMP2     QDMINEVDADGNGTIDFPEFLTMMARKMKDTDSEEEIREAFRVFDKDGNGYISAAELRHV 374

NCaMP7     MTNLGEKLTDEEVGELIREADIDGDGQVNYEEFVQMMTAKGGSGGGSSSRRKWNKAGHAV 357
RCaMP1a    MTNLGEKLTDEEVDEMIREADIDGDGQVNYEEFVQMMTAK-------------------- 442
jGCaMP8    MTNLGEKLTDEEVDEMIREADIDGDGQVNYEEFVQMMTAK-------------------- 422
GCaMP2     MTNLGEKLTDEEVDEMIREADIDGDGQVNYEEFVQMMTA--------------------- 413

NCaMP7     RAIGRLSSMYFADWCVSKKTCPNDKTIVSTFKWAFITDNGKRYRSTARTTYTFAKPMAAN 417
RCaMP1a    ------------------------------------------------------------ 442
jGCaMP8    ------------------------------------------------------------ 422
GCaMP2     ------------------------------------------------------------ 413

NCaMP7     YLKNQPMYVFRKTELKHSKTELNFKEWQKAFTDVMGMDELYK   459
RCaMP1a    ------------------------------------------   442
jGCaMP8    ------------------------------------------   422
GCaMP2     ------------------------------------------   413
```

Figure S1. Clustal Omega multiple sequence alignment of the four sensors. Yellow and blue sequences show CaM binding peptide residues and CaM residues, respectively. Green- and salmon-colored sequences belong to the FP; GFP/mNeongreen and mRuby, respectively. The gray sequence represents the unresolved residues in the crystal structures and were not included in our initial models. Residue indices in our initial models are shifted in the following manner: NCaMP7: *n*-10, RCaMP1a: *n*-37, jGCaMP8: *n*-11, GCaMP2: *n*, with *n* being the residue index in the PDB files.

Table S1. A list of MD simulation runs

System	*Model name*	*Chromophore charge*	*Replicates**
GFP	ON	Anionic	1
mRuby	ON	Anionic	1
mNeongreen	ON	Anionic	1
GCaMP2	Holo	Anionic	1
	Apo	Neutral	1
	Apo*	Neutral	1
RCaMP1a	Holo	Anionic	2
	Apo	Neutral	2
NCaMP7	Holo	Anionic	2
	Apo	Neutral	2
jGCaMP8	Holo	Anionic	2
	Apo	Neutral	2

*For simulation lengths, see Figure S2.

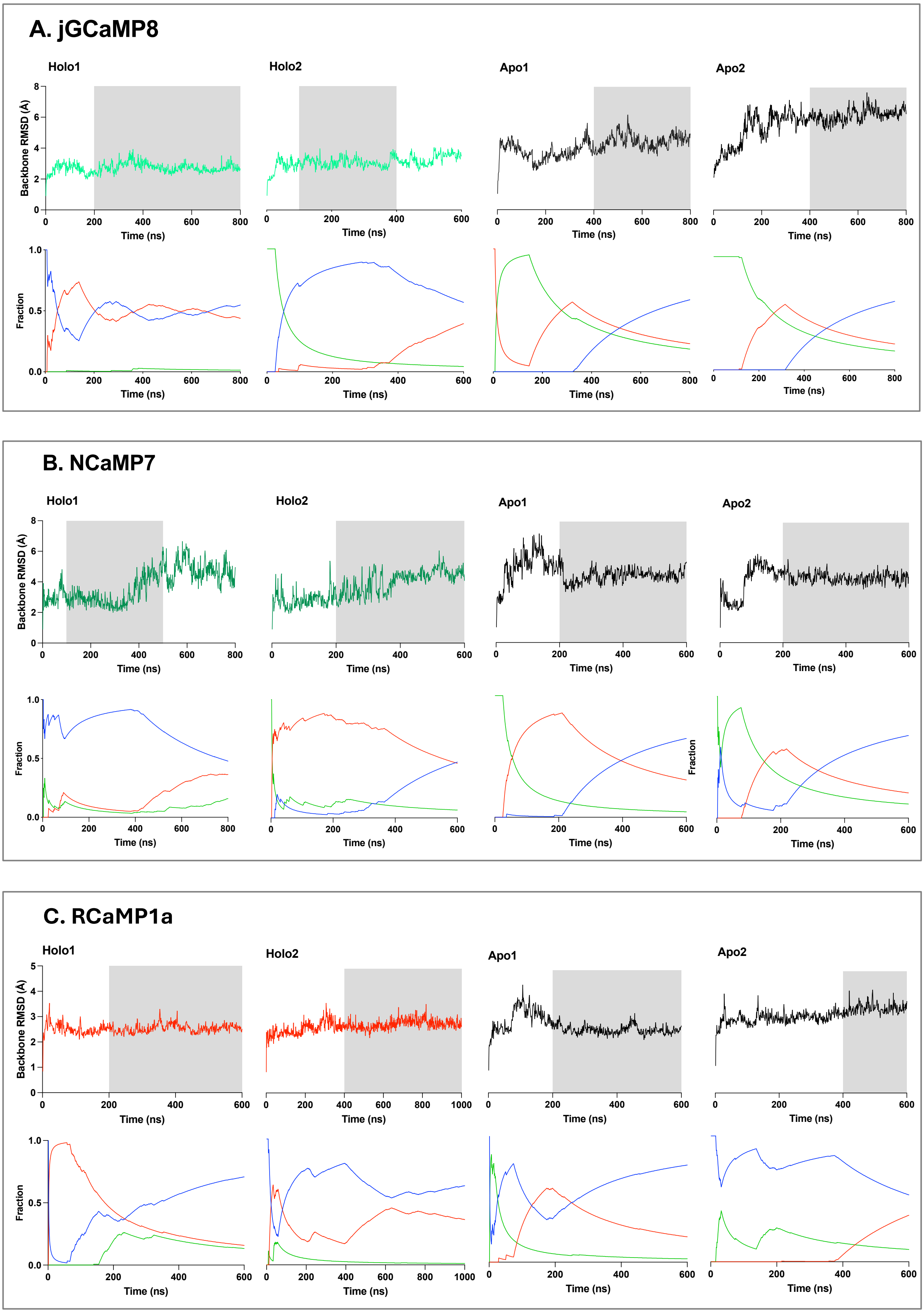
A. jGCaMP8
Holo1
Holo2
Apo1
Apo2
Backbone RMSD (Å)
Time (ns)
Fraction
B. NCaMP7
Holo1
Holo2
Apo1
Apo2
Backbone RMSD (Å)
Time (ns)
Fraction
C. RCaMP1a
Holo1
Holo2
Apo1
Apo2
Backbone RMSD (Å)
Time (ns)
Fraction

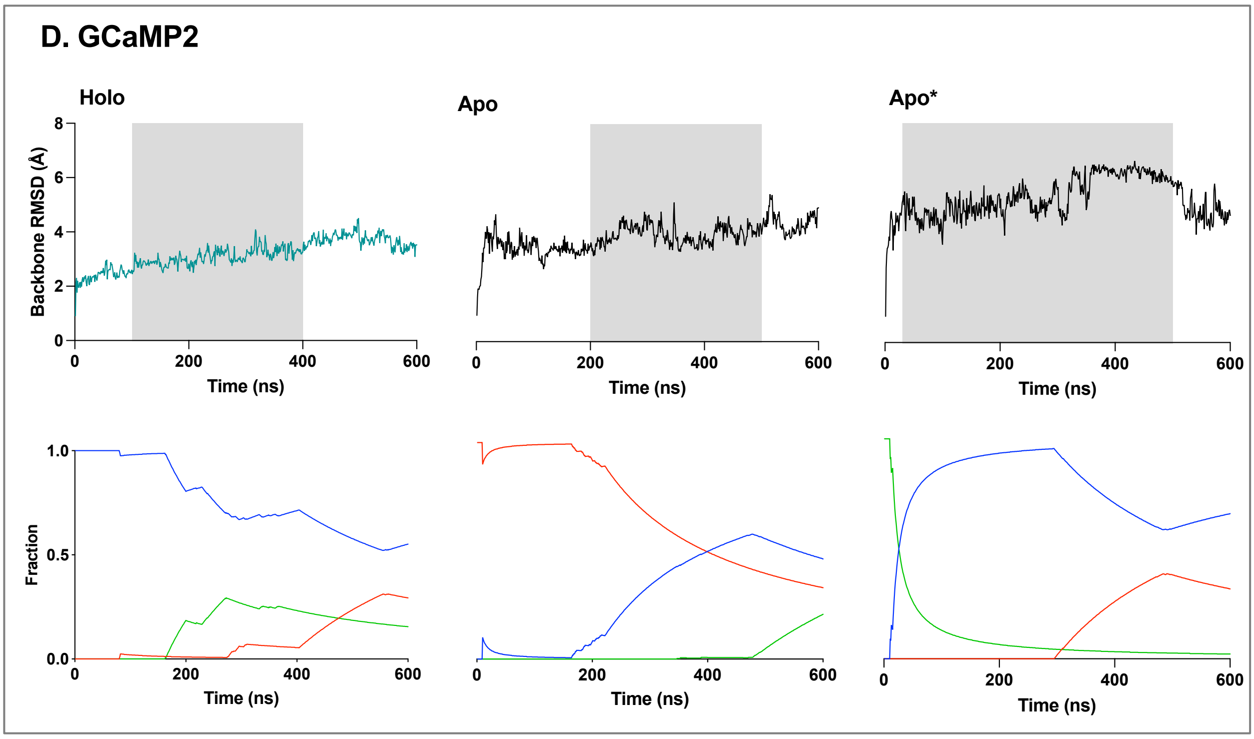

Figure S2. Backbone RMSD (upper) and conformational cluster fraction (lower) plots. Shaded area in RMSD plots indicates the part of the trajectory used in analysis. In cluster fraction vs time plots, each color represents a different a different conformational cluster.

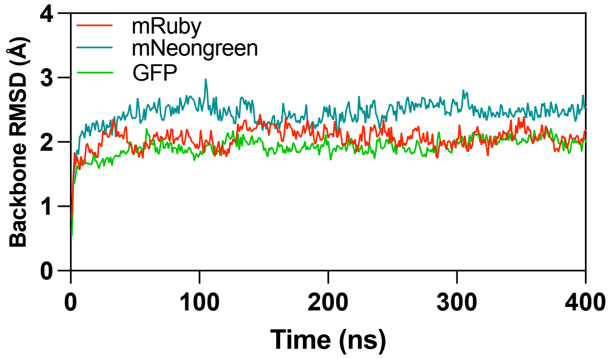

Figure S3. Backbone RMSD of ON state (anionic) parental FPs.

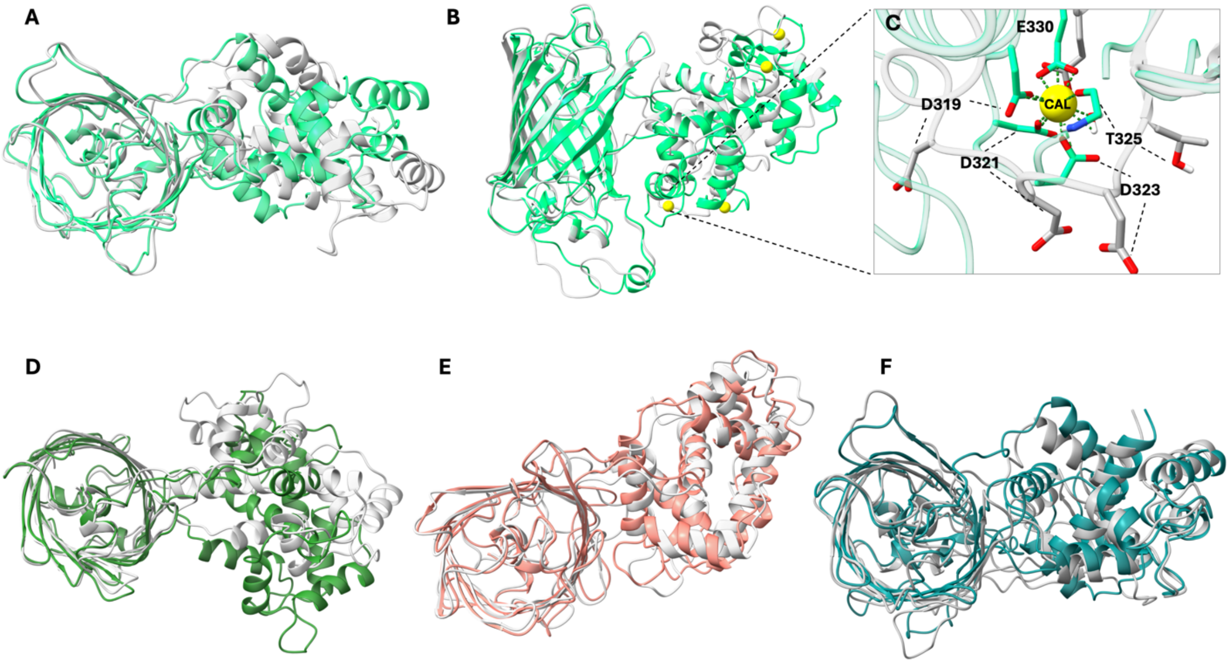

Figure S4. Changing positions of CaM and FP domains relative to each other as a result of calcium removal from the holo sensor. **A-C** jGCaMP8. Top **(A)** and side view **(B)** of *apo* and *holo* states aligned onto the FP domain. **C.** Calcium coordinating residues on the second EF hand motif. Top views of *holo* and *apo* states of **D.** NCaMP7 **E.** RCaMP1a **F.** GCaMP2. *Apo* states are colored silver in each case.

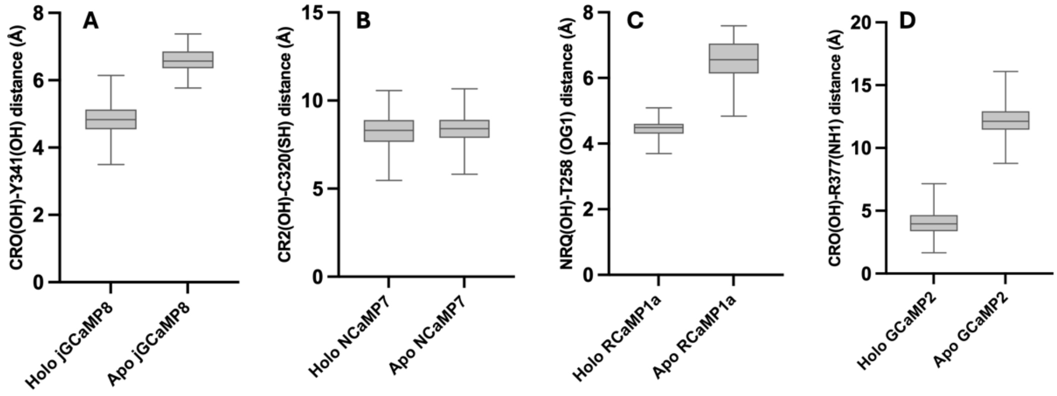

Figure S5. Distribution of interatomic distances between chromophore phenoxy oxygen and its primary hydrogen bond donor in the *holo* (ON) and *apo* (OFF) states. **A.** jGCaMP8; **B.** NCaMP7; **C.** RCaMP1a; **D.** GCaMP2.

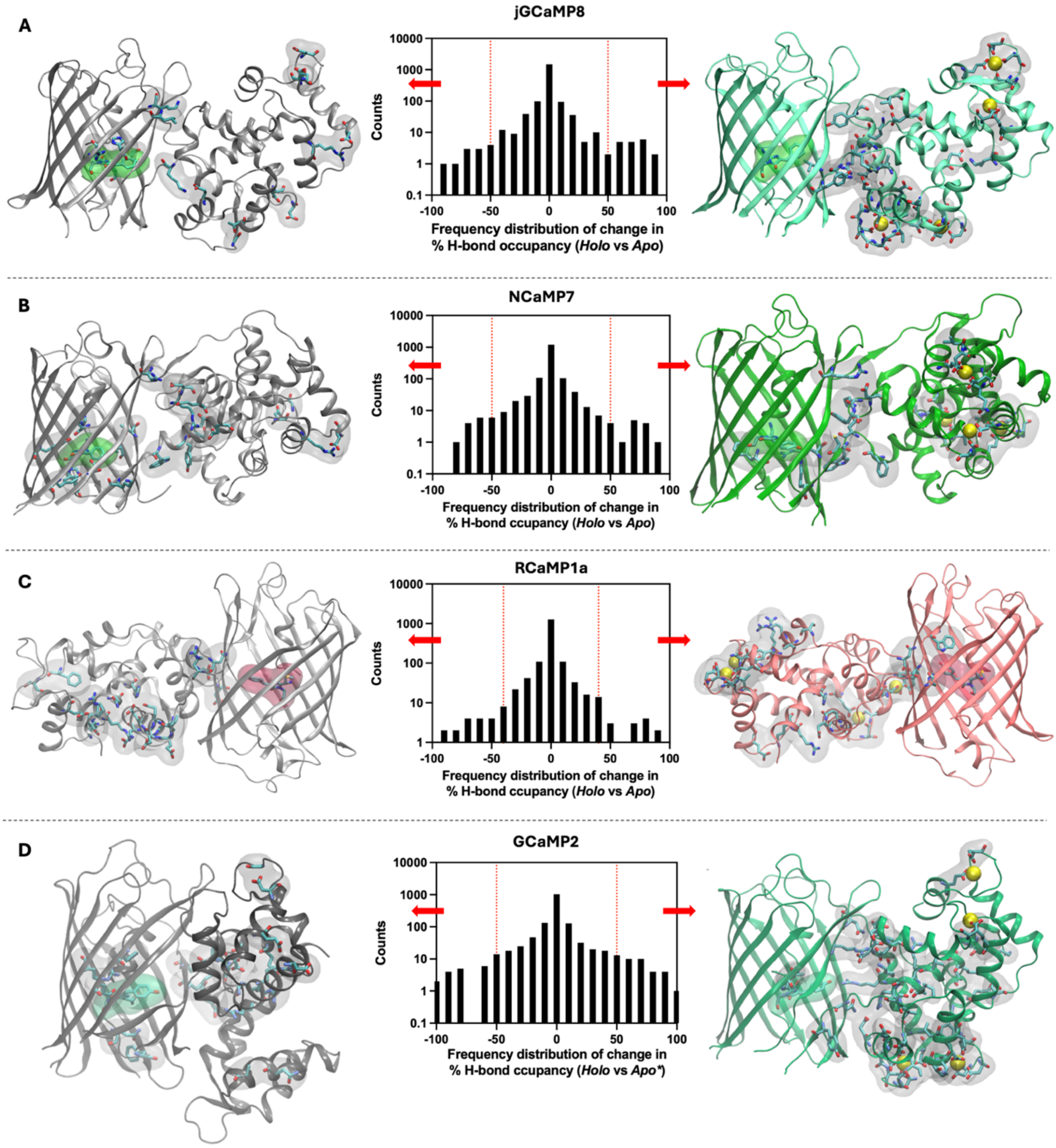

Figure S6. Shift in hydrogen bond occupancies between holo and apo sensors (center). The hydrogen bonds in the regions with > ± 50 % change, marked by the arrows, are mapped on the flanking structures. *apo* states are colored silver; *holo* states are colored based on the color emitted. **A** JGCaMP8; **B** NCaMP7; **C** RCaMP1a; **D** GCamP2. In D, the *apo** system is shown while the *apo* is in Figure 5 in the main text.

Table S2. Progressive change of hydrogen bond occupancies in going from *holo* to *apo** state in GCaMP2

Residue pair	*Location*	*Bond occupancy %*		
		Holo	*Apo*	*Apo**
R81-E387	Interface	96	7	0
E370-A360	CaM	93	9	0
K46-E314	CaM	65	7	0
E317-K46	CaM	89	67	0
D398-E407	CaM	86	18	0
R42-E314	CaM	85	28	0
T382-N83	Interface	79	42	0
E417-K43	CaM	54	0	0
T347-E350	CaM	80	1	21
D323-F319	CaM	69	59	13
R377-D381	CaM	62	0	0
E357-N62	Interface	58	24	0

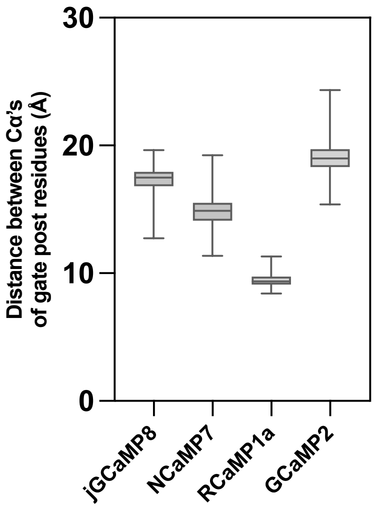

Figure S7. Interatomic distances between C_α atoms of gate post residues in the holo state of each sensor. jGCaMP8: I21-D265, NCaMP7: A135-C320, RCaMP1a: N25-W255, GCaMP2: E61-T303 distance

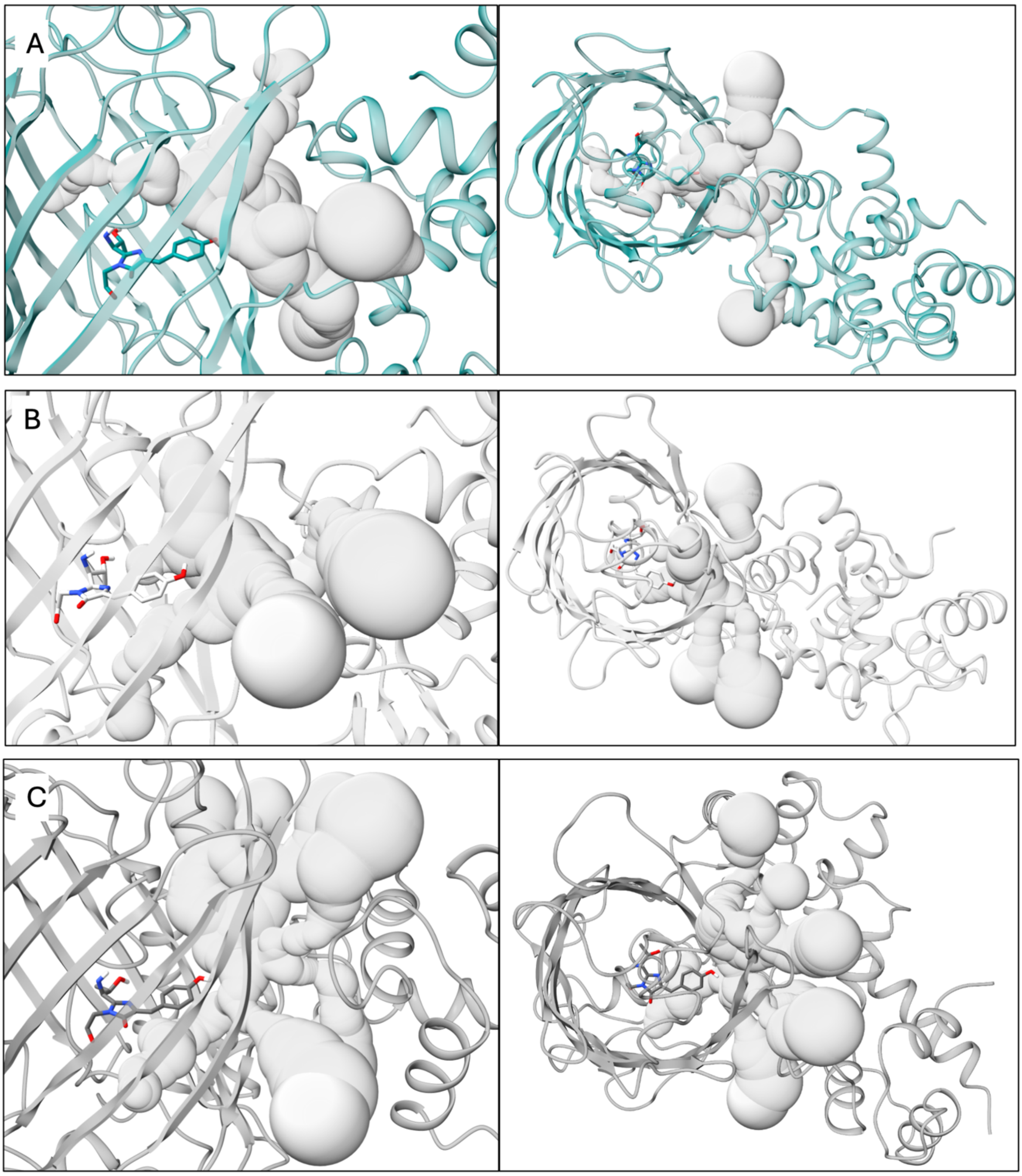

Figure S8 Close up (left) and overall top-down (right) views of representative solvent channels in GCaMP2. **A.** *holo*; **B.** *apo*; **C** *apo**.

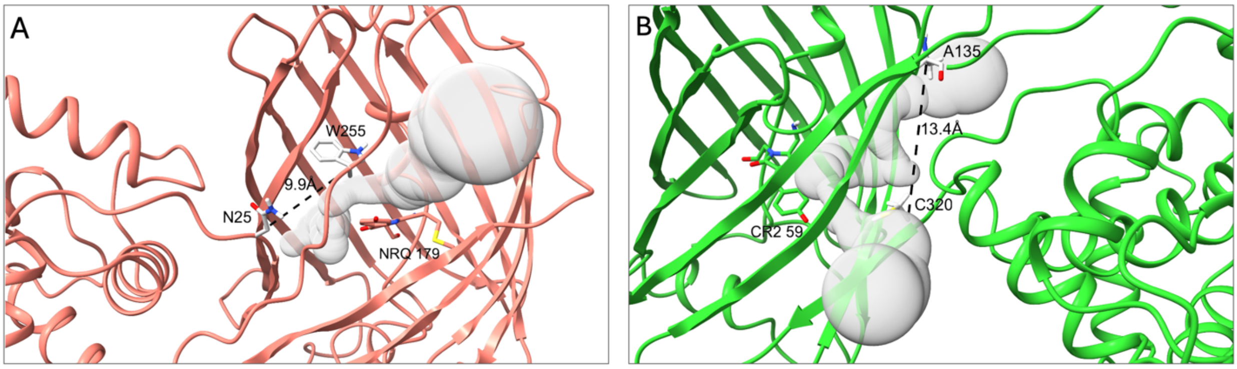

Figure S9 Solvent channels in *holo* **A**.RCaMP1a **B.** NCaMP7

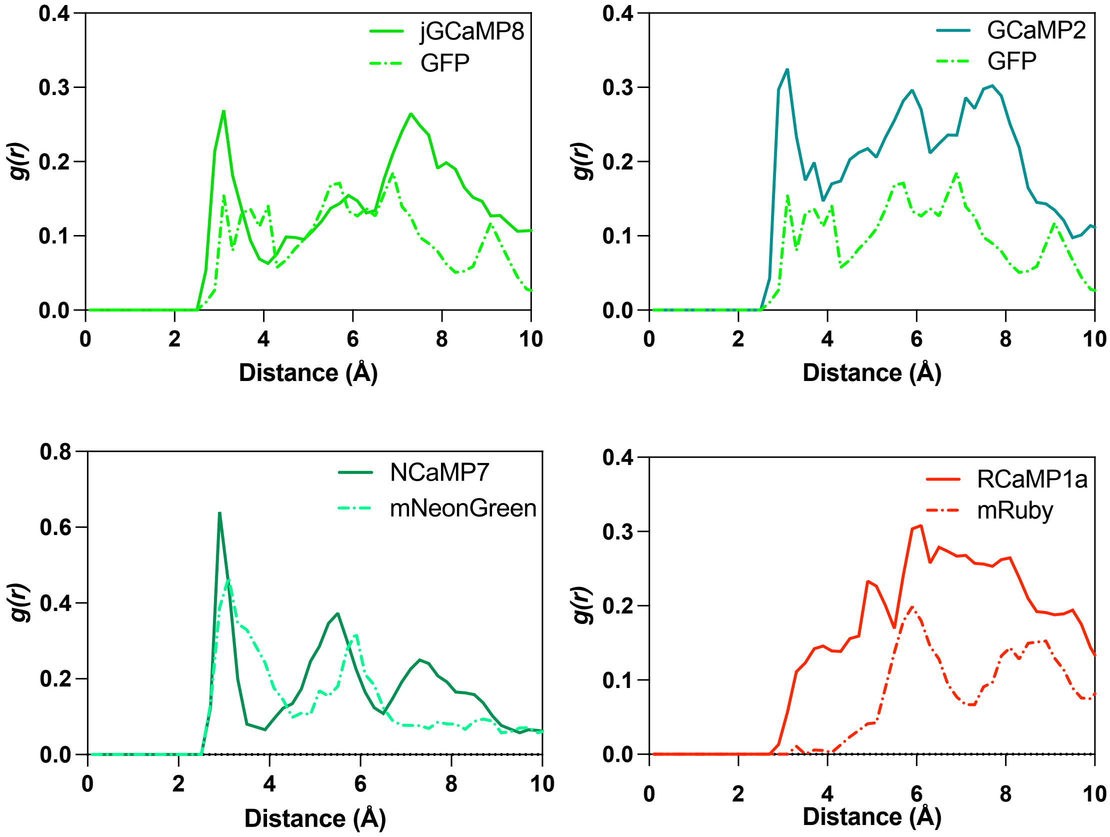

Figure S10. Radial distribution function of water-oxygen within 10 Å of chromophore N2 atom
